# Defining the relationship between infection prevalence and clinical incidence of *Plasmodium falciparum* malaria

**DOI:** 10.1038/ncomms9170

**Published:** 2015-09-08

**Authors:** Ewan Cameron, Katherine E. Battle, Samir Bhatt, Daniel J. Weiss, Donal Bisanzio, Bonnie Mappin, Ursula Dalrymple, Simon I. Hay, David L. Smith, Jamie T. Griffin, Edward A. Wenger, Philip A. Eckhoff, Thomas A. Smith, Melissa A. Penny, Peter W. Gething

**Affiliations:** 1 Department of Zoology, Spatial Ecology and Epidemiology Group, University of Oxford, Tinbergen Building, Oxford OX1 3PS, UK; 2 Wellcome Trust Centre for Human Genetics, University of Oxford, Oxford OX3 7BN, UK; 3 Institute for Health Metrics and Evaluation, University of Washington, Seattle, Washington 98121, USA; 4 Fogarty International Center, National Institutes of Health, Bethesda, Maryland 20892, USA; 5 Department of Infectious Disease Epidemiology, MRC Centre for Outbreak Analysis and Modelling, Imperial College London, London W2 1PG, UK; 6 Institute for Disease Modeling, 1555 132nd Avenue NE, Bellevue, Washington 98005, USA; 7 Department of Epidemiology and Public Health, Swiss Tropical and Public Health Institute, University of Basel, Basel 4002, Switzerland

## Abstract

In many countries health system data remain too weak to accurately enumerate *Plasmodium falciparum* malaria cases. In response, cartographic approaches have been developed that link maps of infection prevalence with mathematical relationships to predict the incidence rate of clinical malaria. Microsimulation (or ‘agent-based') models represent a powerful new paradigm for defining such relationships; however, differences in model structure and calibration data mean that no consensus yet exists on the optimal form for use in disease-burden estimation. Here we develop a Bayesian statistical procedure combining functional regression-based model emulation with Markov Chain Monte Carlo sampling to calibrate three selected microsimulation models against a purpose-built data set of age-structured prevalence and incidence counts. This allows the generation of ensemble forecasts of the prevalence–incidence relationship stratified by age, transmission seasonality, treatment level and exposure history, from which we predict accelerating returns on investments in large-scale intervention campaigns as transmission and prevalence are progressively reduced.

## Abstract (web summary)

Mathematical models are used to predict malaria burden to inform disease control efforts. Here, Cameron *et al*. use Bayesian statistics to calibrate previous models against a data set of age-structured prevalence and incidence, generating stratified forecasts of the prevalence–incidence relationship.

## Graphical abstract

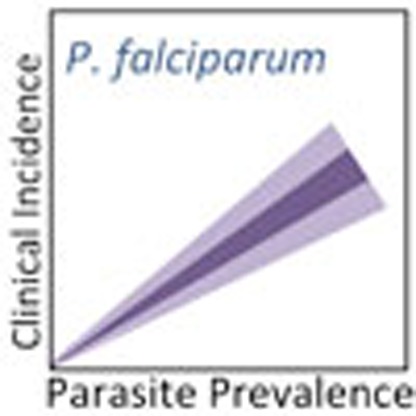

Despite encouraging recent progress, *Plasmodium falciparum* continues to impose an enormous burden of disease and death across sub-Saharan Africa^1^. In many countries with the most intense transmission, disease-reporting infrastructures are weak and precise enumeration of the burden on human health arising from malaria is challenging. This, in turn, limits evidence-based disease-control planning, implementation and evaluation. In response, cartographic approaches have been developed that use maps of infection prevalence (termed the *P. falciparum* parasite rate, *Pf*PR)^2^,^3^ or other transmission metrics^4^ as a basis for estimating the incidence rate of clinical disease in different locations^1^,^5^. While maps of *Pf*PR are becoming increasingly robust, in part because of the proliferation of high-quality data on infection prevalence from nation-wide household surveys, the relationship between *Pf*PR and clinical incidence remains relatively poorly understood and informed by a much smaller and less standardized empirical evidence base.

Recent efforts to construct a suitable *Pf*PR–incidence relationship for *P. falciparum* burden estimation include purely data-driven fits of varying degrees of sophistication from first-order stratification by endemicity class to hierarchical Gaussian process regression^6^,^7^, and projections based on the calibration of a steady-state compartmental transmission model^8^. Over the past decade, a number of sophisticated microsimulation models have been developed that aim to capture all important components of the malaria transmission system, providing a platform to investigate many aspects on the basic epidemiology of the disease and the likely effect of different control strategies^8^,^9^,^10^. Such models simulate infections at the level of distinct individuals within a population, each having experienced a unique history of past exposure and treatment^11^,^12^, and therefore allow inference of the community-level *Pf*PR–incidence relationship. However, conflicts in their predictions arising from differences in the conceptual structures of these models cannot yet be distinguished from those simply because of differences in the data sets used in their calibration, nor indeed from any potential spatiotemporal or ethnic heterogeneity in the underlying relationship. Hence, no consensus yet exists on an appropriate form of the *Pf*PR–incidence curve for use in disease-burden estimation and for addressing other important public-health questions.

The unique potential of microsimulation models for performing detailed epidemiological modelling under realistic conditions^13^ comes at the price of a much greater computational demand than for steady-state models. As a result, the calibration of microsimulation models against empirical data sets has proven a persistent difficulty for applications of these methods across the health sciences^14^, and in particular for malariology^15^,^16^: the common experience being that sophisticated statistical algorithms are required to achieve computational tractability whether the goal is maximum likelihood estimation of model parameters or full posterior inference. To overcome this challenge in the present study we introduce a novel model-emulation procedure on the basis of the technique of functional regression^17^,^18^—in which kernel-weighting methods are used to generate a map from the input space of entomological inoculation rate (EIR) seasonality profile plus model parameter vector to the output space of age-incidence curve plus age-*Pf*PR curve on the basis of a pre-compiled library of noisy, small runtime simulation outputs. The emulator of each model allows fast approximate likelihood evaluations, thereby facilitating thorough posterior sampling under a Markov Chain Monte Carlo (MCMC) algorithm.

In this article we aim to apply the emulator approach to three *P. falciparum* microsimulation modelling frameworks and a standardized calibration data set to define an ensemble model for the *Pf*PR–incidence relationship that incorporates both empirical uncertainty (driven by a limited and noisy calibration data set) and conceptual uncertainty (driven by structural differences between the models). These three frameworks were selected from among the wider family of contemporary mechanistic models on the basis of four criteria: (i) outputs are generated through microsimulation or stochastic transitions through a compartmental structure; (ii) immunity to clinical illness is explicitly modelled; (iii) either software was readily available or the algorithm was sufficiently transparent to replicate the model independently; and (iv) the modelling framework has been extensively documented in peer-reviewed publications. A number of models were identified as satisfying the first criterion with reference to the systematic review of Reiner *et al*.^19^ but were ultimately rejected on the second^13^,^20^, while the third and fourth criteria exclude a minority of codes in development or restricted to proprietary use (for example, the in-house GlaxoSmithKline model for roll-out of the RTS,S vaccine candidate). With the resulting ensemble model we are able to account *during calibration* for (i) the ‘observer effect' (or ‘Hawthorne effect') arising from ethical study designs in which the monitoring campaign itself introduces a treatment rate higher than that previously typical of the target community and (ii) site-to-site differences in the seasonality of malaria transmission. We then further account *on prediction* for (iii) age-, treatment- and seasonality dependence in the *Pf*PR–incidence relationship and (iv) for the effects of recent declines from historically high levels of transmission (and hence exposure-based immunity). From an analysis of these end points, we predict accelerating returns on investments in large-scale intervention campaigns as transmission and prevalence are progressively reduced.

## Results

### Data

The data against which we calibrate each of the three transmission models explored in this study represent a subset of the compilation prepared by Battle *et al*.^21^ in their exhaustive literature review of studies reporting direct measurements of incidence for both *P. falciparum* and *P. vivax* malaria. Here we restricted our focus to those sub-Saharan African *P. falciparum* surveys with active case detection (ACD, where malaria cases are detected in the community) conducted no less frequently than monthly. In a number of these studies, passive case detection (where cases are detected after seeking care at health facilities) was additionally deployed to alleviate missingness from a fraction of febrile episodes occurring entirely between ACD visits. A further constraint imposed was that the incidence observations are available as raw counts with matched person–year observed tallies in at least four distinct age bins. Where incidence observations were presented under multiple case definitions, we select that with a parasite-density threshold, and where multiple thresholds are reported we select that closest to 5,000 parasites per μl. For continuity with previous work^8^ we also included a single passive case detection-only study^22^ that was not otherwise identified by the above criteria. Our final data set is thus composed of measurements from 24 separate studies reporting data for a total of 30 unique sites observed between 1981 and 2011 (Table 1). Contemporaneous, age-structured parasite prevalence data were extracted from the literature to supplement the incidence data for 28 of these sites. Eight of these studies report incidence under a case definition of fever with any detectable parasitaemia (that is, without application of a higher parasite-density threshold designed to improve specificity). In addition, worth noting is that 11 of the 24 studies included in our final data set were not utilized in the previous calibration of the Griffin *et al*. model^8^ (adding 10 unique sites to the 20 used previously).

### Transmission models

The three contemporary transmission platforms employed in this study were OpenMalaria^9^,^23^,^24^,^25^ (run in a single baseline configuration, rather than as full ensemble itself), the EMOD DTK v1.6 (ref. 10, 12, 26, 27, 28) and the Griffin *et al*. model^8^. Here we employ the publicly available microsimulation codes for the former two and, for the latter, a bespoke code based on the compartmental model described therein (we will refer to this implementation as ‘the Griffin IS', that is, Individual Simulation). Each model was run with a 5-day time step under a forced EIR configuration, whereby a pre-determined transmission intensity is imposed as a direct model input, in contrast to ‘full vector' mode simulations in which the EIR is only indirectly controllable through adjustment of ancillary climate and mosquito model parameters. The use of forced EIR here thus is a pragmatic decision to facilitate model fitting at the expense of our ability to capture the dynamic response between host and vector populations (most important at low EIRs) with these simulations. The case management system in each model was configured to yield a 35% probability of effective treatment per febrile episode (formally, per 2-week period with illness in the case of OpenMalaria) during a 90-year period of warm-up simulation time to establish equilibrium levels of immunity under realistic conditions. Here we use the term ‘probability of effective treatment' to mean the direct probability of parasite clearance through drug-based intervention over the course of an illness: that is, the product of a series of steps in the health-care seeking and treatment chain not necessarily modelled explicitly in each code. A year of baseline observations was then sampled before reconfiguration to an 85% probability of effective treatment to simulate the potential ‘observer effect' of an ethical study design at this near-maximal treatment level. The simulation observables here are annual counts of clinical fevers, parasite positives and population size in age bins with end points spaced as {0, 1, 2, 3, 4, 5, 7.5, 10, 15, 25, 35, 45, 60 and 90 years old (y/o)}. Simulations with EIR declining after the warm-up period were effected by direct control, where allowed, by the EMOD DTK and Griffin IS, and through a generic intervention module providing a proportional reduction in the force of infection in the case of OpenMalaria.

### Age dependence of the *Pf*PR_2–10_–incidence relationship

**Figure 1:**
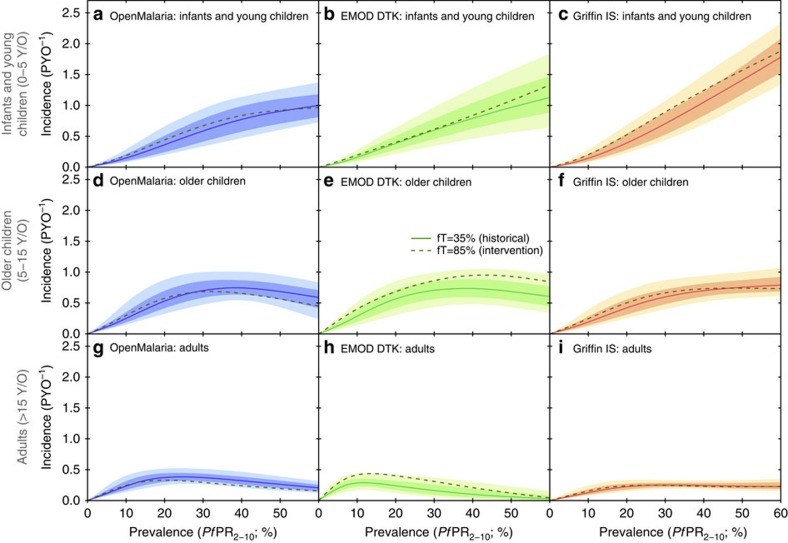
Calibrated posterior predictions of the *P. falciparum* prevalence–incidence relationship under conditions of low historical treatment and low transmission seasonality from the three microsimulation models comprising our ensemble, stratified by age. (**a**,**d**,**g**) OpenMalaria; (**b**,**e**,**h**) EMOD DTK; (**c**,**f**,**i**) Griffin IS. In each panel the coloured curve and shaded zones illustrate the (pointwise) median and surrounding 68 and 95% credible intervals for incidence detectable with daily ACD supposing no change to treatment, while the dashed black lines illustrate the median prediction corresponding to a study year intervention increasing the effective treatment rate from 35 to 85% (that is, the ‘observer effect' of ethical study designs).

Although fitted against a common data set, our posterior calibrations of the three microsimulation models exhibited a number of subtle differences in their predictions for the *Pf*PR_2–10_–incidence relationship stratified by age. Figure 1 presents a direct comparison of their posterior envelopes from simulations under a low seasonality profile (here constant EIR) for three key age groups chosen for consistency with the reporting conventions (and prevalence/incidence-to-mortality modelling methodologies) of the Global Burden of Disease project^29^ and the World Malaria Report^1^: ‘infants and young children' (0–5 y/o exclusive: that is, up to the fifth birthday), ‘older children' (5–15 y/o) and ‘adults' (15+ y/o). Important to note when interpreting these plots is that the prevalence baseline is that for the 2–10 y/o age group (*Pf*PR_2–10_) targeted by the spatiotemporal prevalence maps, to which these curves may be applied for burden estimation^3^. Moreover, the modelled relationships between prevalence and the force of infection are highly nonlinear, so neither should be interpreted naively as a proxy for the other. This is reflected by the convexity in the *Pf*PR_2–10_–incidence curve for infants and young children in the Griffin IS: at low transmission, incidence scales linearly with both EIR and prevalence as each infected individual is unlikely to face an infectious challenge while currently infected; however, as transmission intensity increases, prevalence saturates, whereas super-infection can lead to episodes of clinical disease, hence, the appearance of a faster-than-linear scaling at 20–40% *Pf*PR_2–10_. (This effect is also seen in the original Griffin *et al*. model^8^ fits as highlighted in Supplementary Fig. 9 of our Supplementary Information File.)

As each model implements a similar function for age dependence of the biting rate, the drivers of between-model differences observed here lie primarily in differences between exposure and the development of clinical immunity in each model^8^,^24^,^27^,^30^. In particular, the observation that neither OpenMalaria nor the EMOD DTK exhibits the above-noted convexity in their *Pf*PR_2–10_–incidence curves for infants and young children can be traced to the operation of parts of their exposure-based immunity models on timescales much shorter than those of the Griffin IS. In the latter, the decay timescales of pre-erythrocytic and clinical disease immunity are fixed *a priori* to 10 and 30 years, respectively, which limits the fitting flexibility of these components since reductions to the predicted incidence at young ages are coupled strongly to reductions at older ages. In OpenMalaria, however, the dynamic parasite-density threshold model for clinical illness^23^ has a half-life of just 0.33 years, which enables it to regulate increases in the incidence in infants and young children without forcing the incidence in adults to zero. A similar effect is achieved in the EMOD DTK via the explicit mechanistic simulation of antigenic variation as a modulator of exposure-based immunity.

**Figure 2:**
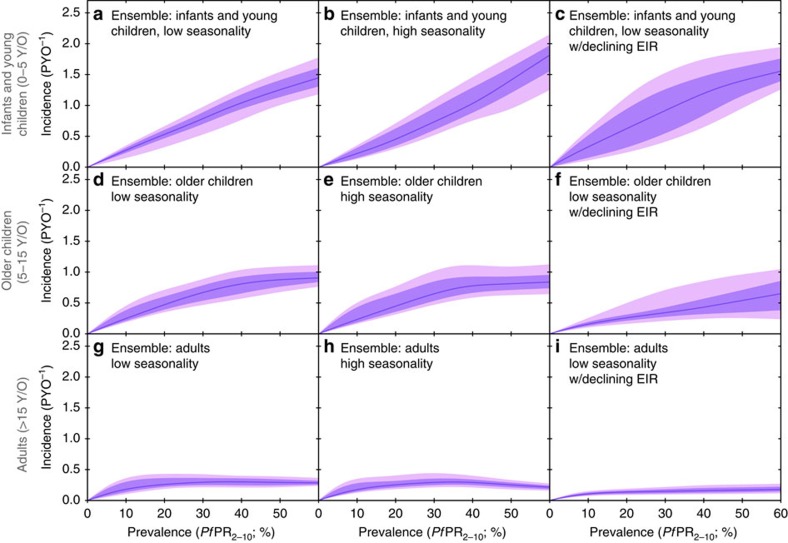
Ensemble model predictions of the *P. falciparum* prevalence–incidence relationship, stratified by age. Predictions are given under conditions of low historical treatment and low transmission seasonality (**a**,**d**,**g**), high seasonality (**b**,**e**,**h**) and low seasonality after a 90% decline in EIR over the past 5 years (**c**,**f**,**i**). In each panel the coloured curve and shaded zones illustrate the (pointwise) median and surrounding 68 and 95% credible intervals for incidence detectable with daily ACD. These ensemble predictions represent a weighted average of the calibrated posteriors from each transmission model with weights assigned by the median of subset posteriors algorithm.

Despite these subtle differences in the *Pf*PR_2–10_–incidence relations predicted by each model at fixed age, there is a strong agreement as to the overall strength of exposure-based immunity in shaping the age dependence of clinical illness from *P. falciparum* malaria: namely, that at low transmission levels corresponding to *Pf*PR_2–10_ levels below 10% the greatest burden is among the adult population, but at higher transmission levels the balance of morbidity quickly shifts towards children (*cf*. refs 8, 23). It is this general agreement we hope to capture in our ensemble predictions, which we produce from a weighted pool of each model's posterior predictive envelopes with weights chosen algorithmically (as described in the Methods under Ensemble Model) to favour two- and three-way agreements. Figure 2 presents our ensemble predictions for the age-structured *Pf*PR_2-–10_–incidence relationships under low seasonality transmission (as shown separately for each model in Fig. 1), as well as for high seasonality transmission and one characterized by a recent decline in transmission intensity.

### Effects of seasonality and a decline in transmission

A comparison between the age-structured *Pf*PR_2–10_–incidence relationships of our ensemble model under conditions of low and high transmission seasonality (the left and middle columns of Fig. 2, respectively) reveals only a modest dependence; most notable is the re-emergence of convexity in the high seasonality curve for infants and young children not seen in the ensemble version at low seasonality. The same trend is observed in the high seasonality simulations presented in Griffin *et al*.^8^ and can be understood as a consequence of the definition of *Pf*PR_2–10_ prevalence used here (and in Griffin *et al*.^8^) as the annualized average: with few parasite-positive cases expected during the long dry season, the relationship between prevalence and transmission intensity is steeper than for the benchmark low seasonality case. Nevertheless, the overall age dependence of the *Pf*PR_2–10_–incidence relationship (that is, the shifting age burden with increasing intensity) is little affected by differences in the seasonality profile. However, in the case that EIR has declined from a historically higher level (illustrated in the right column of Fig. 2 for the scenario of a 90% decline over the past 5 years), the age dependence is notably exaggerated, such that infants and young children bear the majority of the burden at all ages. This effect was readily anticipated, given the presence of a long-lived component (>10 year decay timescale) to exposure-based immunity in all three models. Its quantification here is clearly important for an accurate assessment of burden in the context of recent declines in transmission intensity across much of the African continent^3^.

## Discussion

Through a novel emulator-based approach we have been able to calibrate three contemporary microsimulation models against a common, purpose-built data set of age-structured prevalence and incidence counts across 30 unique sites in sub-Saharan Africa. These calibrations reveal subtle morphological differences between the age-structured *Pf*PR–incidence relationships predicted by each model, but also a general agreement in the age dependence of the burden of clinical illness due to *P. falciparum* malaria at varying levels of transmission. As an ensemble, the combined predictive power of these three models allows the construction of consensus forecasts for the responses of these *Pf*PR–incidence relationships to variations in the seasonality of transmission intensity and the effects of a recent decline in overall EIR. These curves represent a powerful new tool for improving the estimation of malaria disease burden and understanding the implications of changing transmission.

Important to note is that, despite the broad consensus with regard to the expected age distribution of incidence revealed in these ensemble predictions, a substantial degree of uncertainty remains in the overall normalization of the *Pf*PR–incidence relationship owing to the great dispersion observed in total counts between field incidence surveys at different sites with comparable transmission levels. For policy makers considering cartographic burden estimates produced from these curves, it should therefore be emphasized that, although the resulting 95% credible intervals will typically indicate a margin of error of order 33% in the total number of incident cases, corresponding estimates of the proportional change in incidence relative to a given starting year can be made to higher precision, being largely robust against this normalization error. As such, the relative change may allow a more faithful assessment of progress towards elimination than absolute case tallies alone.

**Figure 3:**
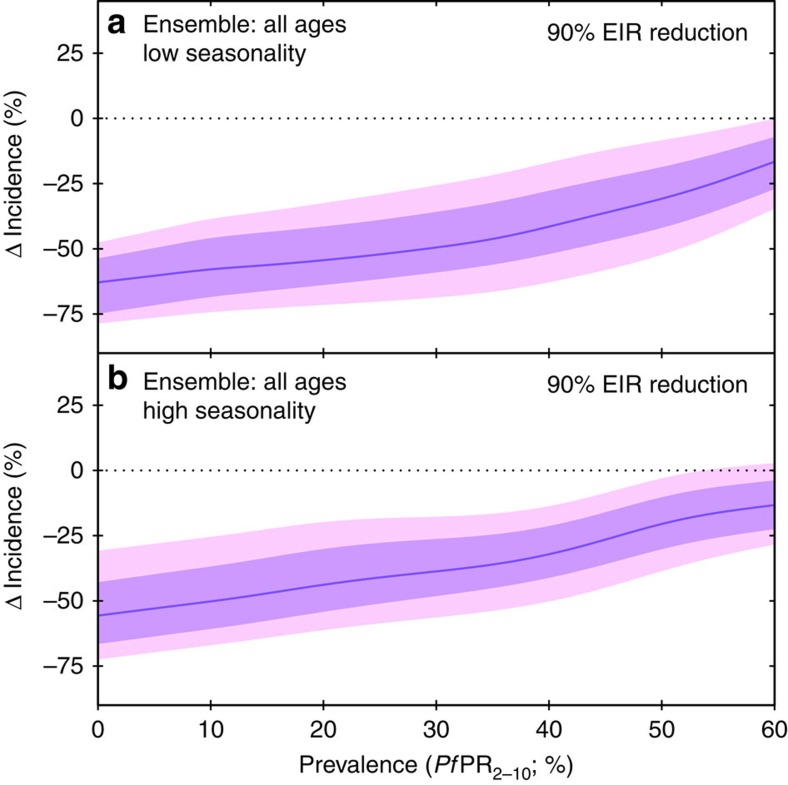
Ensemble model predictions of changes to clinical incidence resulting from a 90% reduction in EIR over the past 5 years, as a function of starting prevalence. In each panel the coloured curve and shaded zones illustrate the (pointwise) median and surrounding 68 and 95% credible intervals for the change in incidence detectable with daily ACD. Predictions are given for low (**a**) and high (**b**) seasonality settings.

The form of the ensemble *Pf*PR–incidence curves presented here also provides a simple insight with profoundly important implications for global malaria elimination and eradication efforts. Figure 3 shows the changes in clinical incidence that our ensemble model predicts for a given fixed reduction in transmission and how this varies depending on the pre-reduction prevalence level. Using the example of a 90% reduction in EIR over a 5-year period, we demonstrate how the proportional reductions in morbidity accelerate as the transmission reduction is applied to progressively lower prevalence settings. In practical terms, this means that a control programme beginning to successfully reduce *Pf*PR in a highly endemic area may initially see only modest improvements in case incidence. However, as intervention coverage continues to scale up and new control measures are introduced, each successive drop in *Pf*PR will yield progressively larger proportional reductions in cases. The origin of this effect lies in the importance of exposure-based immunity for *P. falciparum* malaria, as captured in the microsimulation models explored here, which in turn aim to reproduce the complex relationship between transmission intensity and the age dependence of clinical illness observed in field studies^31^,^32^. While purpose-built microsimulation studies remain essential to estimate the impacts of specific interventions with uncertain efficiency profiles^15^,^33^, our ensemble model demonstrates that for those interventions successful in effecting a general reduction in transmission intensity ever greater pay-offs can be expected as prevalence is brought down progressively across the African continent. This should serve as a rallying call to continue to intensify control efforts that have already yielded substantial declines in infection prevalence and now stand to make increasing impacts on disease burden.

## Methods

### Model emulation

To build a fast emulator for each of the three microsimulation transmission models comprising our ensemble, we adapted a technique from the field of functional data analysis known as functional regression. In this framework we aim to predict the noise-free age-prevalence, *Pf*PR(*a*), and age-incidence curves, *I*(*a*), that would be returned by long runtime (that is, large population) simulations with each model for a given list of input parameters (including the effective treatment rate), **θ**, and annual EIR time series curve, *E*(*t*), using only the noisy age-prevalence and age-incidence curves returned by a reference library of short runtime (small population) simulations. That is, we sought a regression operator,


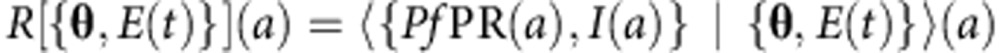


where 〈·|·〉 denotes conditional expectation with respect to the (hidden) stochastic process assumed to generate zero-mean noise in the short runtime output. The nonparametric functional regression solution to this problem^17^ is to construct a kernel-based estimator for *R* in which the output is a (pointwise) mean of functions from the reference library weighted by the ‘distance', *d*(·, ·), of their inputs from those of the target,


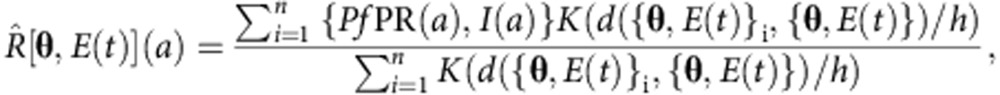


for kernel, *K*(·), and bandwidth parameter, *h*. The intuition here is that the long-run model output for a given target input can be estimated as a weighted mean of the ‘noisy' outputs, with greatest weight given to those of the latter simulated under inputs close to our target.

Following ref. 18 we chose a locally adaptive *k*th nearest neighbours bandwidth, whereby for each input {**θ**, *E*(*t*)} the corresponding *h* was identified such that the distance of the *k*th nearest reference library member was scaled to unity and *K*(·) was set to have unit interval support (here we used the truncated standard Normal). Our distance metric was formed from a weighted combination of two separate metrics: one on the function space of EIR time series and the other on the *p*-dimensional space of input model parameters,


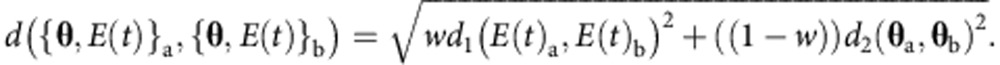


For *d*_1_(·, ·) we chose the logarithmic *L*_2_ distance and for *d*_2_(·, ·) the Mahalanobis distance with diagonal covariance matrix, Ξ, after **θ** was prior integral mass transformed to the unit hypercube. The optimal *k*,*w* and Ξ for each emulator were identified via a downhill gradient search through the space {2^*i*^, [0, 1], [1, *m*]^*m*^*I*} (where *I* denotes the identity matrix and *m* the dimension of the input parameter vector) for the combination maximizing mean predictive accuracy against a training sample of long runtime simulations.

Almost every choice made in the implementation of a functional regression procedure (or, more generally, a kernel-based regression) can potentially have an impact on its predictive performance: (i) the choice of kernel and bandwidth selection procedure^34^, (ii) the choice of distance metric imposed on the input space^17^ and (where relevant, as present) (iii) the design of the reference library. As described above, our approach to the former was to fix the kernel to Normal (Gaussian) and the bandwidth selection to an adaptive *k* nearest-neighbour strategy *a priori*, and to impose a strict form for the distance metric with just a handful of free parameters that we choose iteratively so as to optimize the predictive accuracy of the emulator against a library of long runtime benchmark simulations. However, this procedure can only operate after construction of the reference library of ‘noisy' short runtime simulation output, the design of which we describe next.

### Reference library

The fundamental trade-off in construction of the reference library is between the accuracy of the simulations on which it is built (determined by the simulated population size) and the coverage of input parameter space (determined by the total number of simulations conducted). As a rule of thumb, given that each code is substantially different in its computational overheads: with each microsimulation model requiring up to 90 years of ‘warm-up' time to ensure equilibrium levels of acquired immunity, even simulations with a population of just 200 people can have runtimes in the tens of seconds, while runs with 10,000 people typically take minutes, and runs with 100,000 people tend towards hours. Hence, although larger populations give more stable outputs, it is clearly infeasible to thoroughly populate a library at such runtimes with inputs drawn from a >13-dimensional parameter space. However, indeed, since unbiased estimation is impossible with Nadaraya–Watson-type estimators in the noise-free limit, the reduction of simulation noise to zero would remain undesirable for the purposes of our model emulation, were the computational burdens any less. Through a process of trial and error we eventually settled on population sizes of 5,000 as a suitable basis for building our microsimulation emulators as with this choice it was possible to build libraries of 100,000 realizations spanning densely the input parameter space of each model. For bandwidth optimization and validation (see Supplementary Fig. 1) we produced a further 100-long runtime simulations with a population size of 100,000.

### Transmission code details for openmalaria and EMOD DTK

Numerous aspects of the computational implementation and model structure in both the OpenMalaria and EMOD DTK v1.6 codes have been made extensively customizable to facilitate their application across a diverse range of modelling goals. To ensure the reproducibility of our analysis, we describe here the precise settings used in construction of the reference libraries of simulated age-prevalence and age-incidence curves serving as the reference library in our model emulator.

Our model settings for OpenMalaria (schema version 32) were chosen to follow closely the specification of the ‘base model' described in ref. 15: namely, no decay of immunity (that is, both the ‘IMMUNE_EFFECTOR_DECAY' and ‘ASEXUAL_IMMUNITY_DECAY' parameters set to zero), no mass action effect^35^ of EIR heterogeneity (that is, ‘LOGNORMAL_MASS_ACTION' set to ‘false'), no heterogeneity in treatment seeking or comorbidities and fixed parameters for the age dependence of the biting rate (*S*_∞_=0.049 and E*=0.032). As an exploratory analysis we ran OpenMalaria over a range of EIR levels with zero seasonality for each of the 14 model variants in the best-fit parameterizations described in ref. 15 to trace out approximate *Pf*PR–incidence relationships for each. As only four of these variants (those with the fastest fixed immune decay) exhibited any appreciable difference in this regard, we would broadly expect the results presented herein to be robust against our decision to proceed with the ‘base model' only. To improve the flexibility of the model in representing the diversity of observed age-incidence and age-prevalence counts, we allow the threshold for microscopy-based parasite detection to vary between 20 and 200 parasites per μl in building the reference library.

Where relevant, our model settings for EMOD DTK were then largely chosen in sympathy with those described above for OpenMalaria. In particular, transmission heterogeneity is confirmed zero for MALARIA_SIM mode and we select ‘SURFACE_AREA_DEPENDENT' for the ‘Age_Dependent_Biting_Risk_Type' as the functional form described for this risk profile matches that used in ref. 16. Other key EMOD DTK control option choices here were ‘Enable_Disesase_Mortality' set to zero, ‘Enable_Maternal_Transmission' set to one and ‘Enable_Superinfection' set to one. Again, the parameter controlling the threshold of microscopy-based diagnosis (‘Parasite_Smear_Sensitivity') was allowed to vary over a range equivalent to 20–200 parasites per μl.

For both OpenMalaria and EMOD DTK, the health system settings were simplified to represent administration of a generic antimalarial with the effective treatment rate specified directly as the efficacy (compliance being set to 100%). Simulation of the ‘observer effect' of introducing an enhanced treatment level to a site after years of historically low treatment was implemented via the ‘changeHS' and ‘SimpleHealthTriggeredIntervention' modules, respectively. Specification of EIR seasonality in OpenMalaria was implemented via the ‘fourierSeries' parameterization (discussed further below under Transmission Code Details for EIR Time Series) with a later decline in the mean EIR affected in simulations via the introduction of a generic pre-erythrocytic vaccine intervention (‘vaccineType' set to ‘PEV') blocking a certain fraction of infectious challenges, while in EMOD DTK both these aspects of EIR were specified via the ‘Monthly_EIR' intervention. Finally, it is important to note that each code was run in ‘forced EIR' mode in which the dynamical feedback of transmission intensity between human and vector hosts is turned off. Although potentially less ‘realistic' (being unable to capture the effects of stochastically driven feedback loops between vector and host disease reservoirs), this mode of operation allows for much shorter simulation times, and exploratory analyses with both codes revealed minimal differences in the outputs of interest for EIRs above 0.1 bites per person per year.

### Transmission code details for the Griffin IS

In contrast to the general OpenMalaria and EMOD DTK malaria simulation frameworks, the compartmental model described in ref. 8 features only a single structural form in which (i) immunity decays (with a half-life of *d*_*C*_log(2)=20.8 years for acquired immunity), (ii) transmission is strictly heterogeneous but treatment seeking is not and (iii) the age dependence of the biting rate takes a fixed form (parameters *ρ*=0.85 and *a*_0_=8 years). In the ‘Griffin IS' microsimulation code we developed for this model, the disease states of individuals in a mock population are simulated stochastically using a 5-day time step given the out-of-state transition matrix defined by the equations of Griffin *et al*.^8^ conditioned by their age, past exposure history, and transmission heterogeneity level. After every month of simulated time the population balance is compared with the input template and bins exhibiting a significant discrepancy are resampled and new births added as necessary to maintain a stable demography despite ageing. EIR seasonality and long-term mean declines are imposed directly at each 5-day time step in a manner equivalent to the ‘forced EIR' modes of OpenMalaria and EMOD DTK. The structure of the Griffin IS model so described was found to be well suited to the object-oriented programming paradigm of the c++ language in which we chose to code it, and satisfactory runtimes were easily achieved with help from the GNU scientific library for simulation from parametric probability densities. Extensive comparisons of the output from our microsimulation code against that of the steady-state compartmental version under zero seasonality were performed to validate its behaviour in at least this classical regime.

### Transmission code details for EIR time series

Following the approach of Stuckey *et al*.^36^ we model transmission seasonality as a sinusoidal time series in the logarithm of daily EIR, that is,


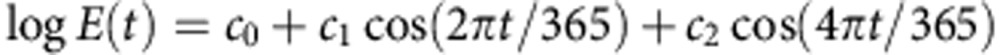


where *c*_1_=*c*_2_=0 corresponds to a constant EIR (no seasonality), *c*_1_≠0, *c*_2_=0 a single peak of seasonal transmission and *c*_1_≠0, *c*_2_≠0a double-peaked profile with half a year between peaks (as seen, for instance, in the monthly EIR time series for Ebolakounou reported in ref. 37). By way of reference we note that for a single-peak seasonal profile (that is, *c*_2_=0) a value of *c*_1_=1 concentrates roughly 75% of transmission within a 6-month period, while a value of *c*_1_=2.5 concentrates the same percentage into just 3 months, equivalent to the definitions of high seasonality previously advocated by Roca-Feltrer *et al*.^38^ and Cairns *et al*.^39^, respectively. We therefore employ the latter (*c*_1_=2.5) as our benchmark for high seasonality posterior prediction and use *c*_1_=0 as our low seasonality benchmark.

When fitting the age-incidence and (where available) age-prevalence data for each site we treat the EIR and its seasonality profile as nuisance parameters, which we integrate out (stochastically) via our MCMC algorithm. For this purpose we suppose the following priors:


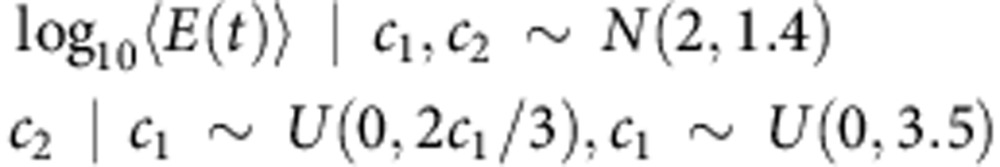


that is, we restrict *c*_2_ to be no more than two-thirds the value of *c*_1_, and given the resulting seasonality profile we draw its mean EIR from a broad log-Normal distribution centred on an EIR of 100.

An important question for the modelling of seasonality in this context is whether or not the seasonal profile is indeed identifiable, given only age-structured incidence counts as data and no site-specific prior information concerning the annual EIR time series. An analysis of our posterior inferences for Ndiop and Dielmo (1990–1993) suggests the affirmative as our estimates of EIR=63 (15–120 (95% CrI)) and *c*_1_=0.9 (0.2–2.6), and EIR=313 (100–670) and *c*_1_=0.5 (0–1.5), respectively, are comparable to their contemporary estimates of EIR=20, with transmission restricted to the brief rainy season in Ndiop and EIR=200 with year-round transmission owing to the presence of a nearby river in Dielmo^40^,^41^. In principle, one might also seek to allow for a diversity of long-term historic changes in transmission during fitting; however, aside from identifiability concerns, the computational requirements to build a well-sampled emulator in this case could become excessive. It is also worth noting, as a caveat to our simulations of the ‘observer effect' of treatment, that by running these microsimulation codes in forced EIR mode we cannot capture any follow-on effect of treatment itself reducing transmission (through reduced human infectiousness to mosquitoes). This may lead to a slight overcompensation for the ‘observer effect' in our fits; however, we judged this preferable to neglect the issue.

### Model calibration

With our model emulator able to provide rapidly a near approximation to the long runtime limit {*Pf*PR(*a*), *I*(*a*)} for each transmission model belonging to any given {**θ**, *E*(*t*)} pairing, the remaining requirements for posterior exploration were specification of a likelihood function for the observed data given the model, and specification of priors on the input parameters. For the former we introduced a hierarchical Bayesian structure allowing for both site-level random effects and overdispersion in the data, with these and the annual EIR time series treated as nuisance parameters. With *y*_*ijk*_ denoting the observed incidence in the *k*th incidence age bin of the *j*th site in the *i*th study, and *p*_*ijm*_ the observed prevalence in the *m*th prevalence age bin of the same (where available; for all but two sites),


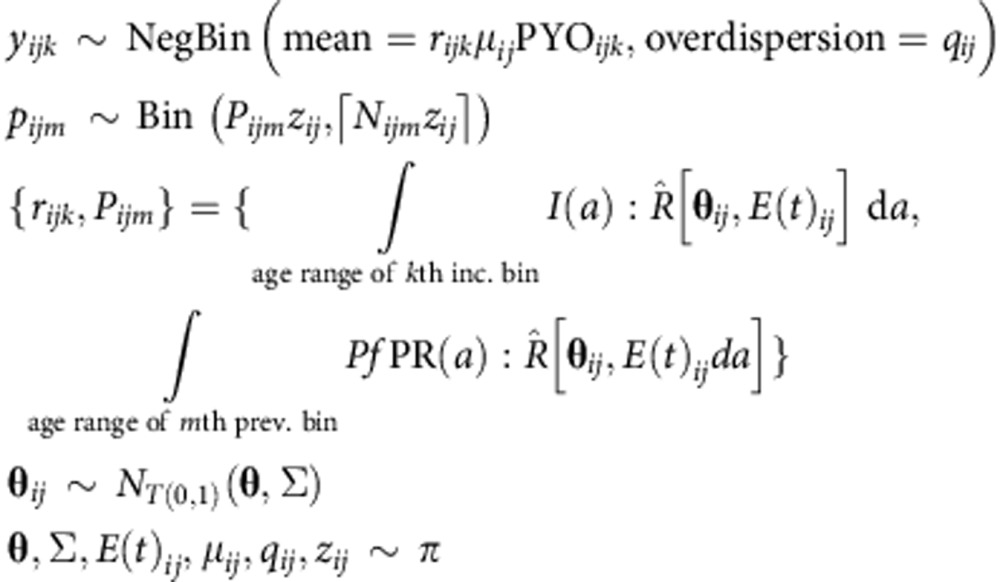


where *r*_*ijk*_ represents the expected (long runtime) incidence rate in the given incidence age bin, and *P*_*ijm*_ the expected prevalence in the given prevalence age bin, approximated via the emulator for the transmission model being fit. The likelihood was completed through prior densities (represented here by the place holder, *π*) on the expected model parameters and site-specific EIR time series, along with the site-specific random effects, *μ*_*ij*_, and overdispersion terms, *q*_*ij*_ and *z*_*ij*_, described below under random effect and overdispersion priors.

Although an investigation of the posterior predictives for the age-incidence and age-prevalence data on a site-by-site basis under each emulator confirms that the adopted noise model is sufficiently flexible to account for the observational variance here, visual inspection of the discrepancy between model and data in the age-incidence (Supplementary Figs 3, 5 and 7) and age-prevalence (Supplementary Figs 4, 6 and 8) plots for some of these sites (for example, Ngerenya and Kenya) reveals a degree of ‘structural noise' (that is, a limitation of the models to reproduce the observed age dependencies). This issue, also noted in previous studies^8^,^23^, likely follows primarily from discrepancies in the levels of heterogeneity in exposure and case management between model and site^42^, although one might hypothesize that unmodelled spatial variation in the underlying transmission dynamics (for example, variation in vector species^43^ or parasite genetic diversity^44^) could also play a role. Both issues warrant future investigation when further ACD-based incidence studies increasing the coverage of countries in Central and Southern Africa (currently under-represented in our data set) become available.

Rather than performing joint MCMC sampling of the complete space of this statistical model—which is of dimensionality >100 as it comprises the full set of nuisance parameters for each study, location and year observed in addition to the global model parameters of interest (**θ**) and their site-specific random realizations (**θ**_*ij*_)—we instead performed MCMC (with simulated tempering^45^ to improve mixing) on each site separately and combined these to approximate the full posterior with importance sample reweighting^46^. The feasibility of this approach is in part facilitated by the weakness of the parameter constraints imposed by the data from each site individually, although *en masse* the full data set is ultimately strongly informative with regard to particular parameters from each model. With the magnitude of the observed incidence rate commonly held as a far less reliable indicator of the ground truth than the shape of its age dependence^47^, and the magnitude of the predicted incidence in each model similarly sensitive to assumptions regarding the maximum duration of a single illness event, our prior on the site-specific random effect allowing for incidence scaling, *μ*_*ij*_, was deliberately made broad and minimally informative. As a result, a secondary phase of normalization is required to achieve concordance between our model parameter posteriors and the average magnitude of observed counts at each site, which we implement via a linear regression of the expected-to-observed all-age incidence ratios against the frequency of ACD for each draw from our full posterior (as described further under Normalization and Calibration to Daily ACD). All statistical computations were performed in the R environment^48^.

### Parameter priors for transmission model parameters

Each of the three microsimulation codes comprising our ensemble requires the user to specify values for numerous controlling parameters. Previous studies with OpenMalaria^15^,^23^,^24^,^25^,^30^ and EMOD DTK^10^,^12^,^27^,^28^ have sought to constrain these in an essentially stepwise manner through comparisons against disjoint data sets targeting specific segments of each model, whereas the Griffin *et al*. model has been calibrated only in its steady-state (non-microsimulation) form^8^ against a homogeneous age-incidence data set including a significant fraction of the studies used here (Table 1). To allow each model a comparable degree of flexibility in the present analysis, we adopt deliberately broad priors on the parameters of all three codes with respect to the constraints suggested by previous analyses. In the Supplementary Discussion (see Supplementary Information) we examine the impact on the posterior predictive *Pf*PR_2–10_–incidence curves of returning to some of the previously fit or ‘default' values of the key OpenMalaria and EMOD DTK parameters.

Our calibration of OpenMalaria includes 14 free parameters assigned priors of the following forms typically matching the mean but at least doubling the 95% CI ranges quoted in previous papers^9^,^23^,^24^,^25^,^30^: *S*_imm_ (Beta), *γ*_p_ (log-Normal), 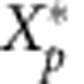 (Gamma), 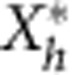 (log-Normal), 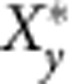 (Gamma), 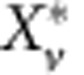 (log-Normal), *a*_*m*_ (Beta), 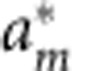 (Gamma), 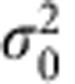 (Normal), *α* (log-Normal), 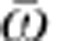 (log-Normal), 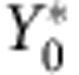 (log-Normal), 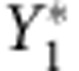 (log-Normal) and 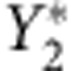 (log-Normal). Our calibration of EMOD DTK includes 13 free parameters assigned priors to the following forms chosen primarily to concentrate mass near the ‘default' values suggested in the EMOD documentation: ‘Antigen_Switch_Rate' (log-Normal), ‘Clinical_Fever_ Threshold_High' (uniform), ‘Clinical_Fever_Threshold_Low' (uniform), ‘Falciparum_MSP_Variants' (Poisson), ‘Falciparum_PfEMP1_variants' (Poisson), ‘Maternal_Antibody_Protection' (Beta), ‘Maternal_Antibody_Decay_Rate' (log-Normal), ‘MSP1_Merozoite_Kill_Fraction' (Beta), ‘MSP2_Merozoite_Kill_Fraction' (Beta), ‘Pyrogenic_Threshold' (log-Normal), ‘Falciparum_Nonspecific_Types' (Poisson), ‘Max_Individual_Infections' (Uniform) and ‘Nonspecific_Antigenicity_Factor' (log-Uniform). Finally, our calibration of the Griffin IS includes 19 free parameters assigned priors of the following forms, roughly matching the 95% prior credible intervals of Griffin *et al*.^8^: *d*_U_ (log-Normal), ID_0_ (log-Normal), *κ*_D_ (Normal), *u*_D_ (log-Normal), *b*_0_ (Beta), IB_0_ (log-Normal), *κ*_B_ (log-Normal), *u*_B_ (log-Normal), *φ*_0_ (Beta), *φ*_1_ (Beta), IC_0_ (log-Normal), *κ*_C_ (log-Normal), *u*_C_ (log-Normal), *P*_M_ (Beta), *d*_M_ (log-Normal), *d*_1_ (Beta), *f*_D0_ (Beta), *a*_D_ (log-Normal) and *γ*_D_ (log-Normal).

### Random effect and overdispersion priors

To complete our hierarchical Bayesian model for the observed data set of age-structured incidence and prevalence counts described above (under Model Calibration), we must add priors for the distributions of site-specific random effects and overdispersions to those on the EIR time series parameters given earlier. Inspection of the fits presented in previous work^8^ to calibrate the steady-state version of their transmission model led us to expect both marked variations in normalizations of the observed age-incidence and age-prevalence relations at a similar EIR (that is, a potentially large random effects term) and (occasionally) marked structural departures from the model (that is, potentially large overdispersion terms). Hence, we allowed broad priors on each of the form:


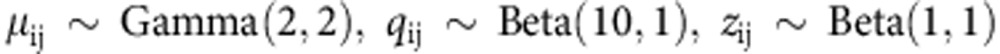


where the Gamma distribution takes the shape–rate parameterization. These specifications give an expectation of one for the random effect term in a given survey (corresponding to no rescaling of incidence) and a weak overdispersion of 0.91 (close to the Poissonian limit of one) in the case of incidence, but both allow wide variation about these means if the data so demands; the overdispersion term acting on the observed prevalence, *z*_*ij*_, is given greater freedom as the sample sizes of prevalence surveys are typically much larger than those of incidence, increasing the potential for structural conflict between the model and data. Finally, to ensure that our posterior inferences regarding the mean parameterization, **θ**, are not unnecessarily weakened by the assumed variance on the site-specific realizations, **θ**_*ij*_, unless the data strongly favour this outcome for a certain parameter, we place an Exponential prior on Σ of mean 0.01. Important to note is that we have not at this stage adopted a specific model for the influence of ACD frequency on the observed incidence counts. Instead, we allow any contribution of this form to be absorbed into our fitted random effect terms, only re-calibrating our predictions to daily ACD during our subsequent normalization step (as described below).

### Normalization and calibration to daily ACD

To facilitate exploration of the parameter posterior for each model we specified above only a weak prior on the random effects term, *μ*_*ij*_, scaling the overall incidence of each site, nor do we explicitly include a covariate representing the frequency of ACD in our likelihood function, although one might expect this to be an important contributor to between-study variance. Hence, we instead perform a further stage of analysis to normalize our emulator posterior the prediction of incidence observable via daily ACD. We implement this calibration through a simple regression model to infer the mean ratio between total incidence observed and model predicted at daily ACD via a simple linear regression against the logarithm of ACD period for those studies applying a parasite threshold in their case definition (assumed to improve specificity). This normalization is performed on each posterior draw; a useful illustration is that shown in Supplementary Fig. 2: for each model we have extracted the mean and s.d. of *μ*_*ij*_ at each site and performed a joint linear regression against the logarithm of ACD period in which the slope is shared across models, while the intercept is not. The resulting plot highlights the relatively small fraction of the observational variance explained by differences in the ACD period, although (as expected) there is a general trend towards surveys conducted at longer ACD follow-up periods to yield lower incidence estimates (by a factor of order 2).

### Posterior prediction

By drawing from the MCMC output representing the parameter posterior for each transmission model, and re-running our model emulator across a range of mean EIR levels, we are able to construct the posterior predictive curves for the *Pf*PR–incidence relationship in each age group under a given seasonality profile and treatment history. We therefore chose a representative template for low seasonality (constant EIR) and high seasonality (sinusoidal in log EIR with a factor of 7.3 variation between maximum and minimum; *cf*. ref. 38) and generated posterior predictive curves for each seasonality and treatment history. A further library of posterior predictive curves was then generated using the emulator approach with an additional set of noisy input simulations from each transmission model under the scenario of a 90% decline in the mean EIR over the past 5 years. This accounts for immunity acquired at historically higher transmission levels when forecasting incidence at sites with recent success in scaling up interventions.

### Ensemble model

The canonical Bayesian approach to ensemble prediction under multiple competing models in the Bayesian paradigm is that of model averaging^49^, in which a weighted average of the posterior predictives is formed with weights proportional to the marginal likelihoods of the models under consideration. However, although attractive for their perceived ‘Occam's Razor'-like penalization of model complexity, marginal likelihoods are potentially highly sensitive to the parameter priors assigned to each model^46^,^50^, and in this case we have limited prior information to inform our choices (as discussed above under Parameter Priors). As such, we did not attempt Bayesian model averaging in this case. Nevertheless, rather than defaulting simply to an equal weighting for each model, we would prefer to reward consistency between the predictions of these competing models (following the paradigm of ‘weighting by agreement' identified in a recent review^51^), such that where two agree with similar credible intervals but the third does not, we up-weight the former two relative to the latter. To achieve this effect with a quantitative, reproducible algorithm we adopted the ‘M-posteriors' routine^52^; although originally described for combining posterior samples under equal partitions of a single observed data set fit with a single model, the version described therein of Weiszfeld's algorithm for constructing a median of point measures with kernel-based discrepancy distance provides exactly the functionality we required here. Worth noting is that in this setting (that is, location of the empirical posterior median distribution) the problem is one of convex optimization to which the Weiszfeld algorithm ensures a stable solution^53^. A caveat to this general scheme for ensemble construction is that one may over-reward ‘group-think' where model structures have not been arrived at completely independently—although we note that substantial differences are evident in both the conceptualization and actualization of each microsimulation model considered in this study. Unlike in Bayesian model averaging, the weights assigned to each model here are different for each separate set of posterior predictive curves (low/high seasonality, low/high treatment and so on). Testament to the overall consistency of these three models, it is to be noted that no model is ever assigned less than an 18% contribution to the ensemble by the M-posteriors algorithm.

## Additional information

**How to cite this article:** Cameron, E. *et al*. Defining the relationship between infection prevalence and clinical incidence of *Plasmodium falciparum* malaria. *Nat. Commun*. 6:8170 doi: 10.1038/ncomms9170 (2015).

## Supplementary Material

Supplementary Information Supplementary Figures 1-9, Supplementary Discussion and Supplementary References

## Figures and Tables

**Table 1:** Overview of the prevalence–incidence data set used for model calibration.

Study	Site, Country	Year (s)	*Pf*PR_2–10_	Treat.	*τ*_ACD_*	Threshold
Ba *et al*.^54^†	Ndiop, Senegal	1993	0.26	0.639	1	>3,600 p μl^−1^
Bloland *et al*.^55^†	Asembo Bay, Kenya	1992	0.79	0.870	14	Age specific
Bonnet *et al*.^37^	Koundou, Cameroon	1997–1998	0.72	0.807	1	>1,000 p μl^−1^
	Ebolakouno, Cameroon	1997–1998	0.66	0.807	1	>1,000 p μl^−1^
Bougouma *et al*.^56^,^57^†	Saponé, Burkina Faso	2007	0.67	0.770	3	>2,500 p μl^−1^
Coulibaly *et al*.^58^†	Bandiagara, Mali	1999	0.29	0.944	7	Any patent
Diallo *et al*.^59^,^60^	Dakar, Senegal	1996–1997	0.014	0.495	7	Any patent
	S. Dakar,	1994	0.003	0.495	7	Any patent
Dicko *et al*.^61^	Donéguébougou, Mali	1999–2000	0.403	0.944	7	Any patent
	Sotuba, Mali	1999–2000	0.086	0.944	7	Any patent
Fillol *et al*.^62^†	Niakhar, Senegal	2003	0.22	0.820	7	>3,000 p μl^−1^
Greenwood *et al*.^63^†	Farafenni, The Gambia	1981–1982	0.32	0.682	30	Age specific
Guinovart *et al*.^22^	Manhiça, Mozambique	2003–2005	0.20	0.820	·	Any patent
Henry *et al*.^64^	Katiola, Côte d'Ivoire	1997–1998	0.91	0.686	1	Age specific
	Korhogo, Côte d'Ivoire	1997–1998	0.88	0.686	1	Age specific
	Korhogo, Côte d'Ivoire	1997–1998	0.83	0.686	1	Age specific
Loha *et al*.^65^†	Chano Mille, Ethiopia	2009–2011	0.044	0.812	7	Any patent
Lusingu *et al*.^66^	Mgome, Tanzania	2001	0.91	0.850	30	Age specific
	Ubiri, Tanzania	2001	0.27	0.850	30	Age specific
	Magamba, Tanzania	2001	0.067	0.850	30	Age specific
Molez *et al*.^67^†	Barkedji, Senegal	1994–1995	0.098	0.443	10	Age specific
Mwangi *et al*.^68^,^69^	Ngerenya, Kenya	1999–2001	0.25	0.870	7	>2,500 p μl^−1^
	Chonyi, Kenya	1999–2001	0.41	0.870	7	>2,500 p μl^−1^
Nebie *et al*.^70^†	Balonghin, Burkina Faso	2003	0.63	0.722	1	>5,000 p μl^−1^
Owusu-Agyei *et al*.^71^	Kintampo, Ghana	2004	0.72	0.922	2.33	>5,000 p μl^−1^
Rogier *et al*.^72^	Dielmo, Senegal	1990	0.89	0.639	1	Sharp increase
Saute *et al*.^73^	Manhiça, Mozambique	1996–1999	0.26	0.384	7	Any patient
Schellenberg *et al*.^47^	Ifakara, Tanzania	2000–2001	0.19	0.850	7	Any patient
Thompson *et al*.^74^	Matola, Mozambique	1992–1995	0.38	0.526	1	Age specific
Trape *et al*.^75^†	Linzolo, Republic of Congo	1983–1984	0.79	0.788	1	p/leu. >2
Trape *et al*.^40^	Dielmo, Senegal	2007–2008	0.20	0.950	2.33	p/leu. >3.5
Velema *et al*.^76^†	Pahou, Benin	1989	0.51	0.661	30	>1,000 p μl^−1^

ACD, active case detection; *Pf*PR, *P. falciparum* parasite rate.

^*^Here *τ*_ACD_ denotes the period of ACD in days for each study. Note also that in the Threshold column p μl^−1^ stands for parasites per microlitre, and p/leu. the ratio of parasites to leucocytes.

^†^Highlights studies not included in the previous Griffin *et al*.^8^ model calibration.

^·^Symbol denotes the one study here that did not conduct ACD, but was included here for consistency with the previous analysis of Griffin *et al*.
