# Supplementary material for: Defining the relationship between infection prevalence and clinical incidence of Plasmodium falciparum malaria
Source: Nat Commun. 2015 Sep 8;6:8170. doi: 10.1038/ncomms9170 (PMC4569718; doi:10.1038/ncomms9170)
Supplement: Supplementary Information — Supplementary Figures 1-9, Supplementary Discussion and Supplementary References [file ncomms9170-s1.pdf]

## Supplementary Figures

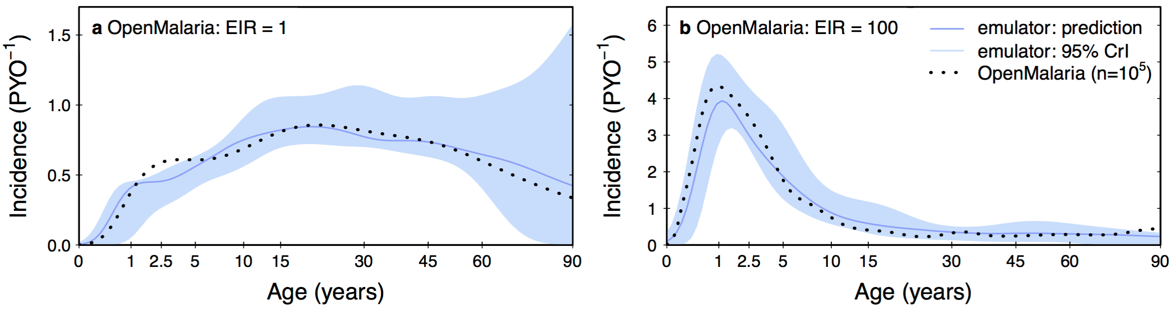


**Supplementary Figure 1: Predictive accuracy of the functional regression-based model emulator illustrated through a comparison against the benchmark age–incidence curves from two long runtime simulations of OpenMalaria.** The OpenMalaria outputs shown as dotted lines in each case are recovered from "micro-simulations" with 100,000 individuals under the "base model" parameterisation of Smith *et al.*[2] with (**a**) a constant (zero seasonality) EIR profile at a mean of 1 IBPPY and (**b**) 100 IBPPY. The dark blue lines indicate our (median) emulator predictions under the same inputs built via functional regression with a 16 nearest neighbour kernel running on a library of "noisy" OpenMalaria outputs from runs with 5000 individuals; the surrounding light blue shaded areas trace out the corresponding 95% credible intervals (pointwise).

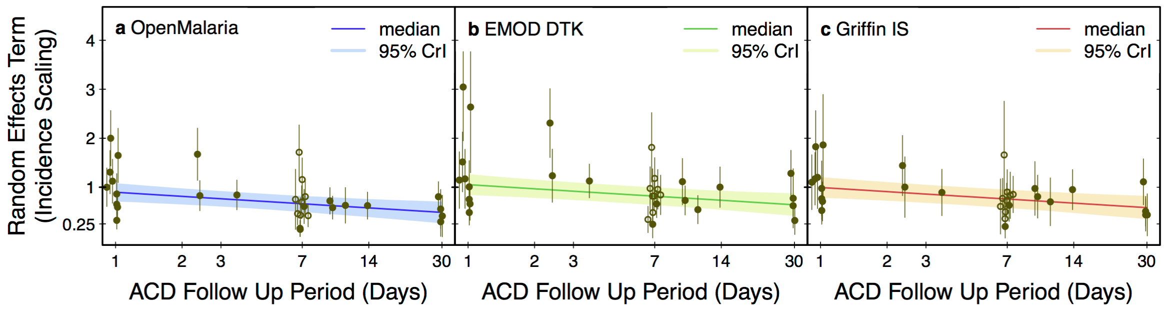


**Supplementary Figure 2: Dependence of the site-specific random effects term scaling total incidence on the frequency of ACD.** The posterior mean and standard deviation of the random effects term for each site is marked with a circle and (95% CrI) error bar: dark grey, filled circles distinguish studies applying a specific parasite density threshold in their case definitions from those that do not (light grey, open circles). The posterior median and (pointwise) 95% credible interval from a linear regression of the random effects (from the density thresholding subset) against log ACD period fitted jointly in slope for all three models is illustrated by the coloured lines and shaded areas on each panel.

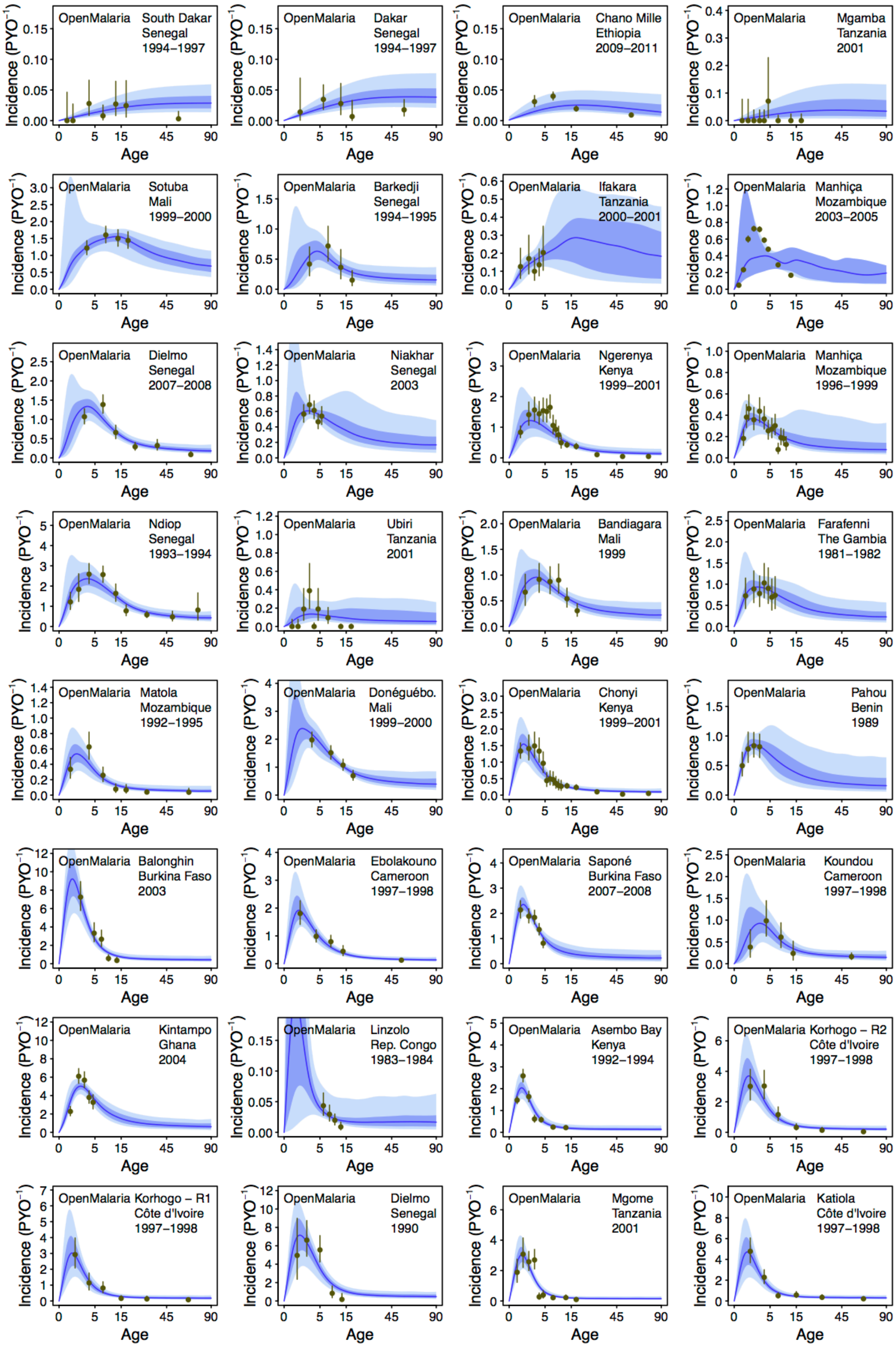


**Supplementary Figure 3: Fitted age–incidence curves from emulation of the OpenMalaria code constrained in model parameter space at site-specific treatment levels.** The coloured lines and areas shown represent the (pointwise) median plus 68% and 95% credible intervals, while the black dots and error bars indicate the available data and its 95% uncertainty under a Poisson likelihood (adopted for illustrative purposes only).

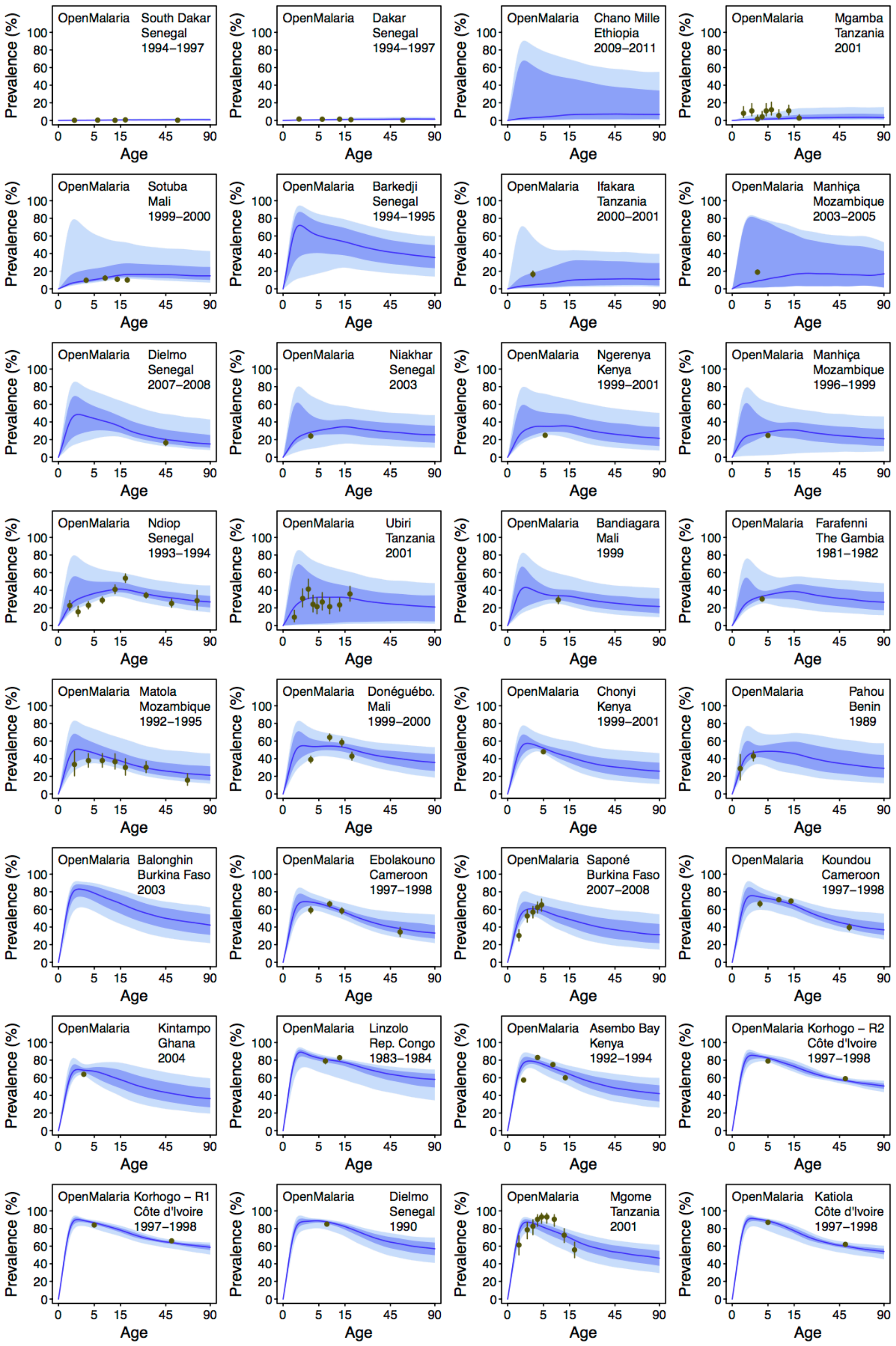


**Supplementary Figure 4: Fitted age–prevalence curves from emulation of the OpenMalaria code constrained in model parameter space at site-specific treatment levels.** The coloured lines and areas shown represent the (pointwise) median plus 68% and 95% credible intervals, while the black dots and error bars indicate the available data and its 95% uncertainty under a Binomial likelihood.

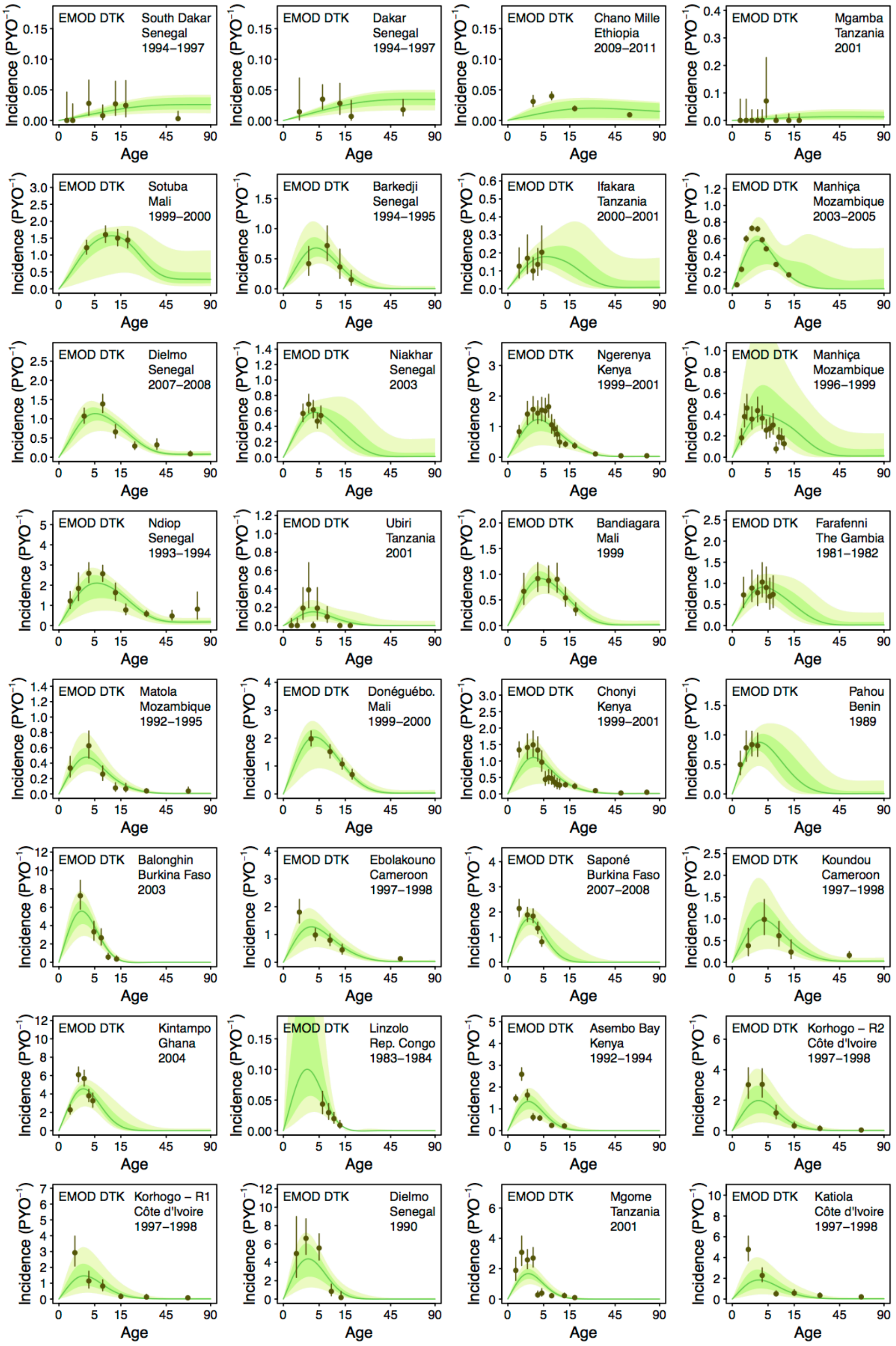


**Supplementary Figure 5: Fitted age–incidence curves from emulation of the EMOD DTK code constrained in model parameter space at site-specific treatment levels.** The coloured lines and areas shown represent the (pointwise) median plus 68% and 95% credible intervals, while the black dots and error bars indicate the available data and its 95% uncertainty under a Poisson likelihood (adopted for illustrative purposes only).

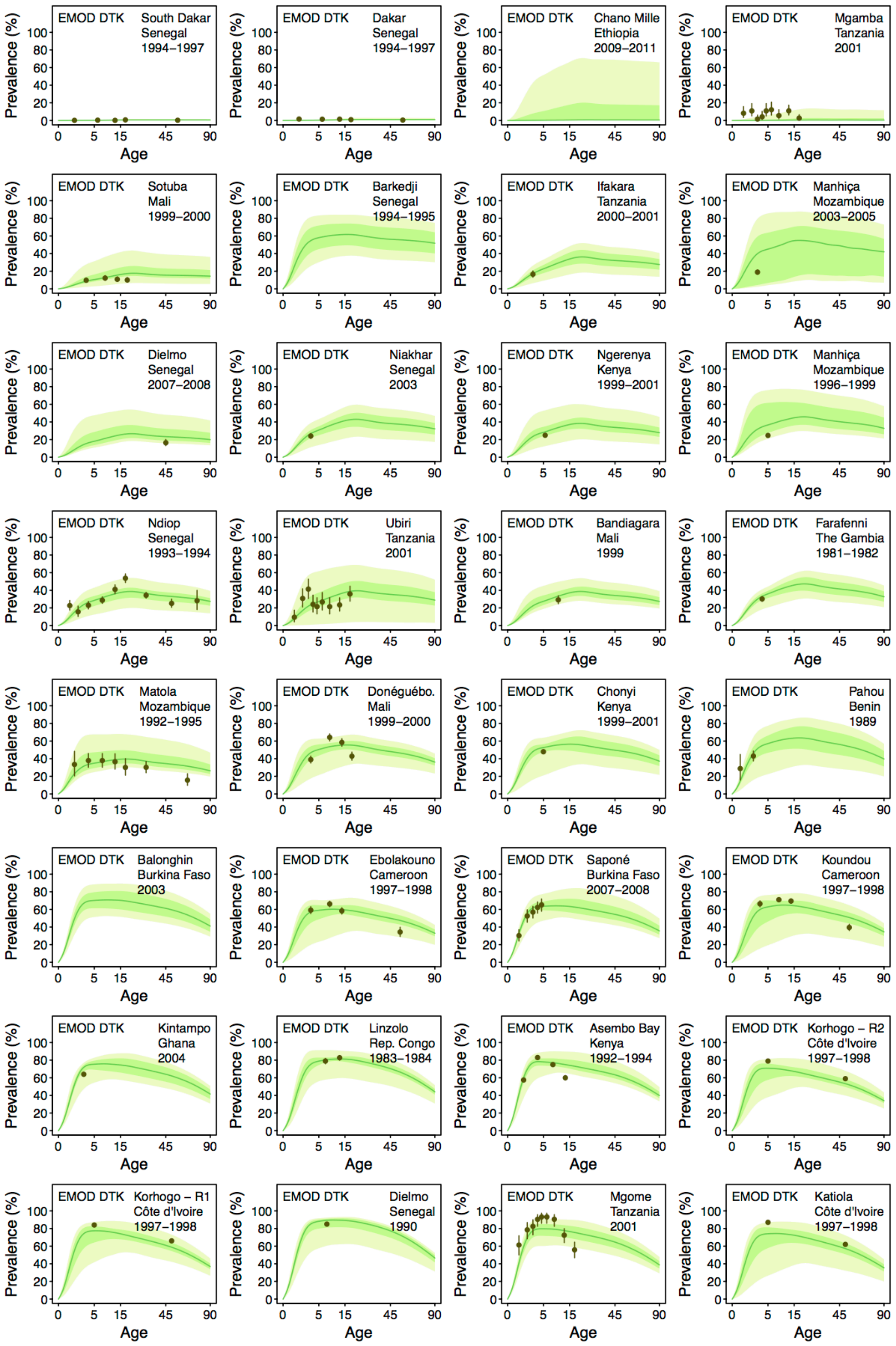


**Supplementary Figure 6: Fitted age–prevalence curves from emulation of the EMOD DTK code constrained in model parameter space at site-specific treatment levels.** The coloured lines and areas shown represent the (pointwise) median plus 68% and 95% credible intervals, while the black dots and error bars indicate the available data and its 95% uncertainty under a Binomial likelihood.

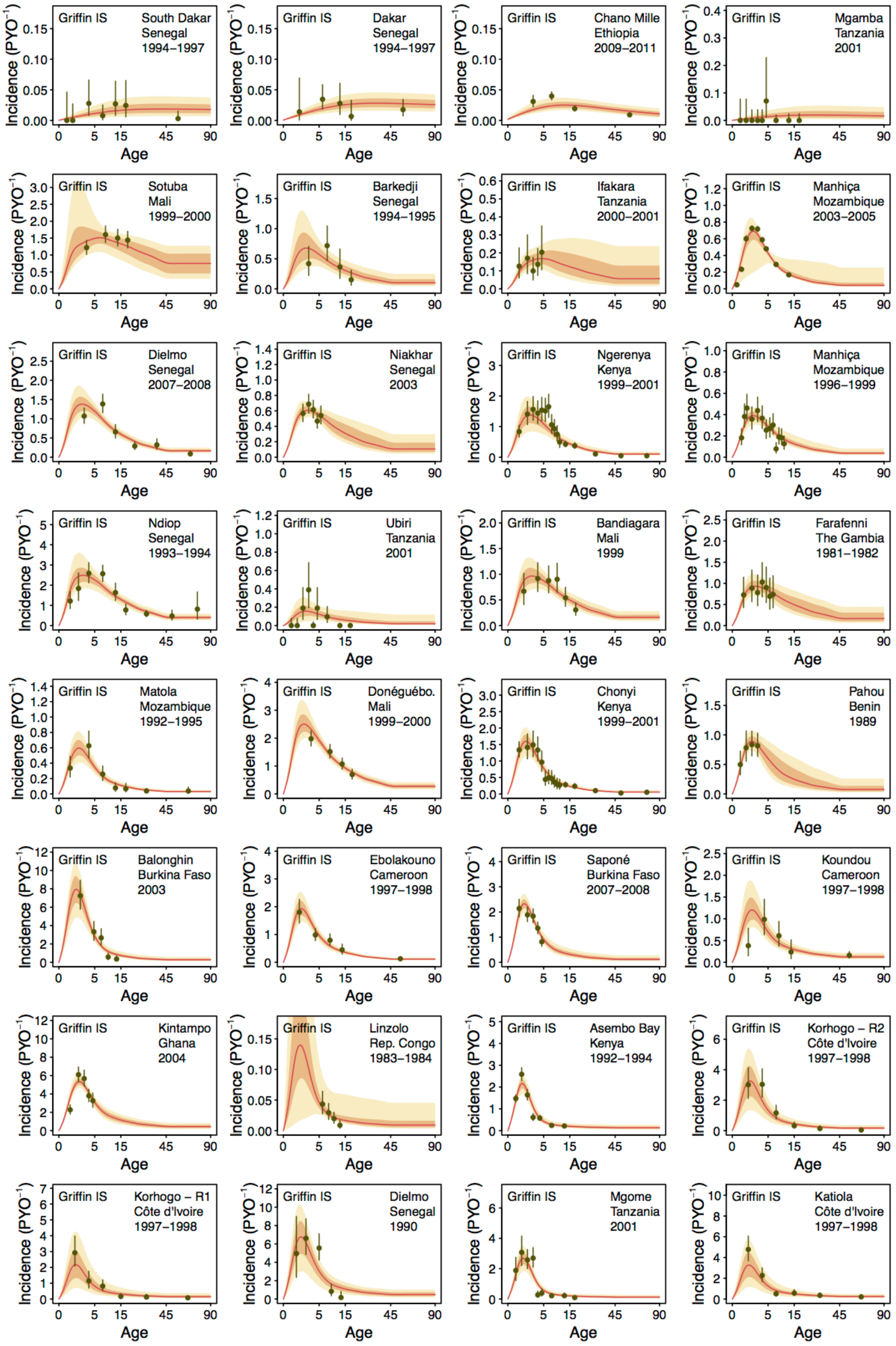


**Supplementary Figure 7: Fitted age–incidence curves from emulation of the Griffin IS code constrained in model parameter space at site-specific treatment levels.** The coloured lines and areas shown represent the (pointwise) median plus 68% and 95% credible intervals, while the black dots and error bars indicate the available data and its 95% uncertainty under a Poisson likelihood (adopted for illustrative purposes only).

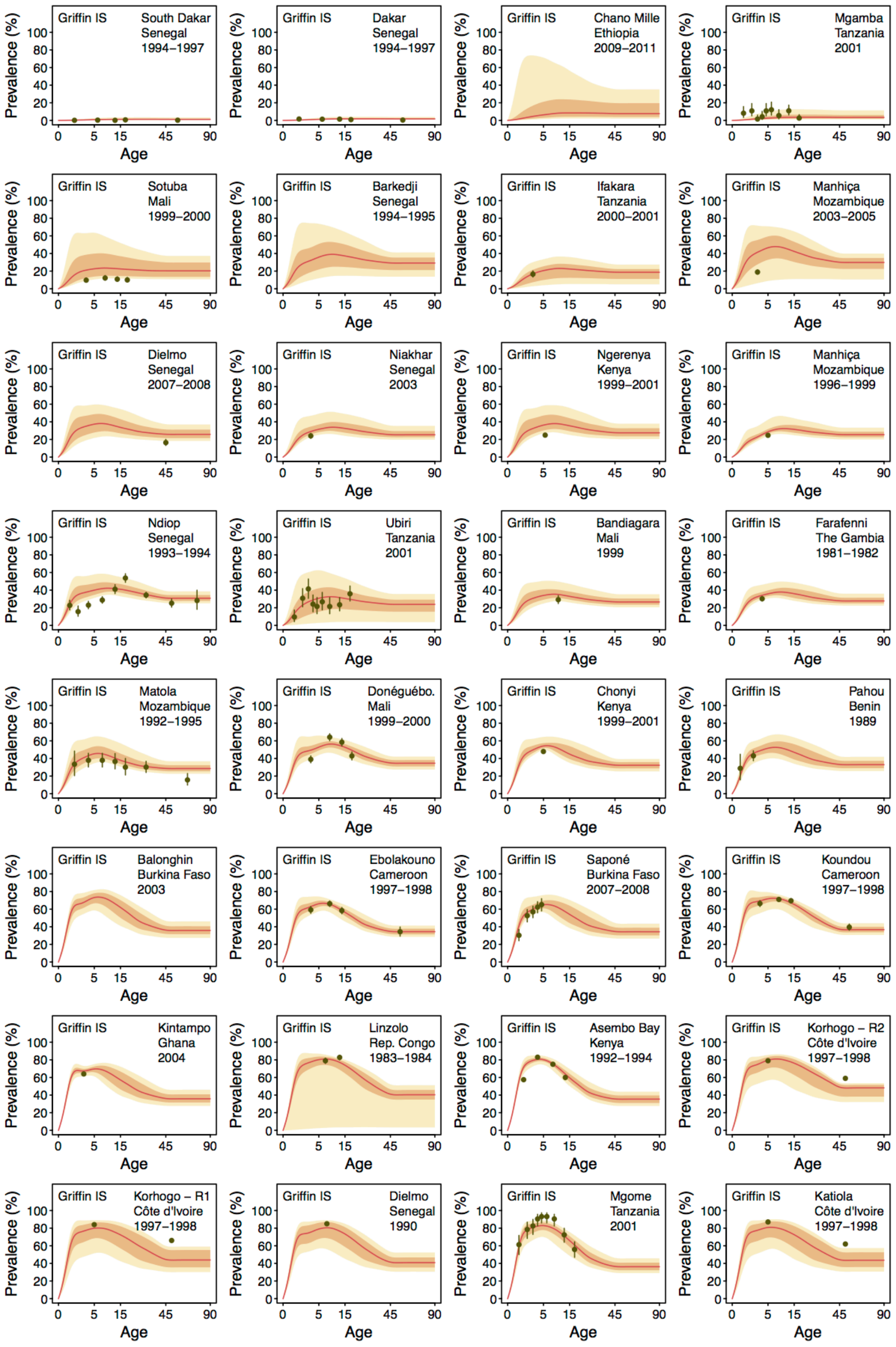


**Supplementary Figure 8: Fitted age–prevalence curves from emulation of the Griffin IS code constrained in model parameter space at site-specific treatment levels.** The coloured lines and areas shown represent the (pointwise) median plus 68% and 95% credible intervals, while the black dots and error bars indicate the available data and its 95% uncertainty under a Binomial likelihood.

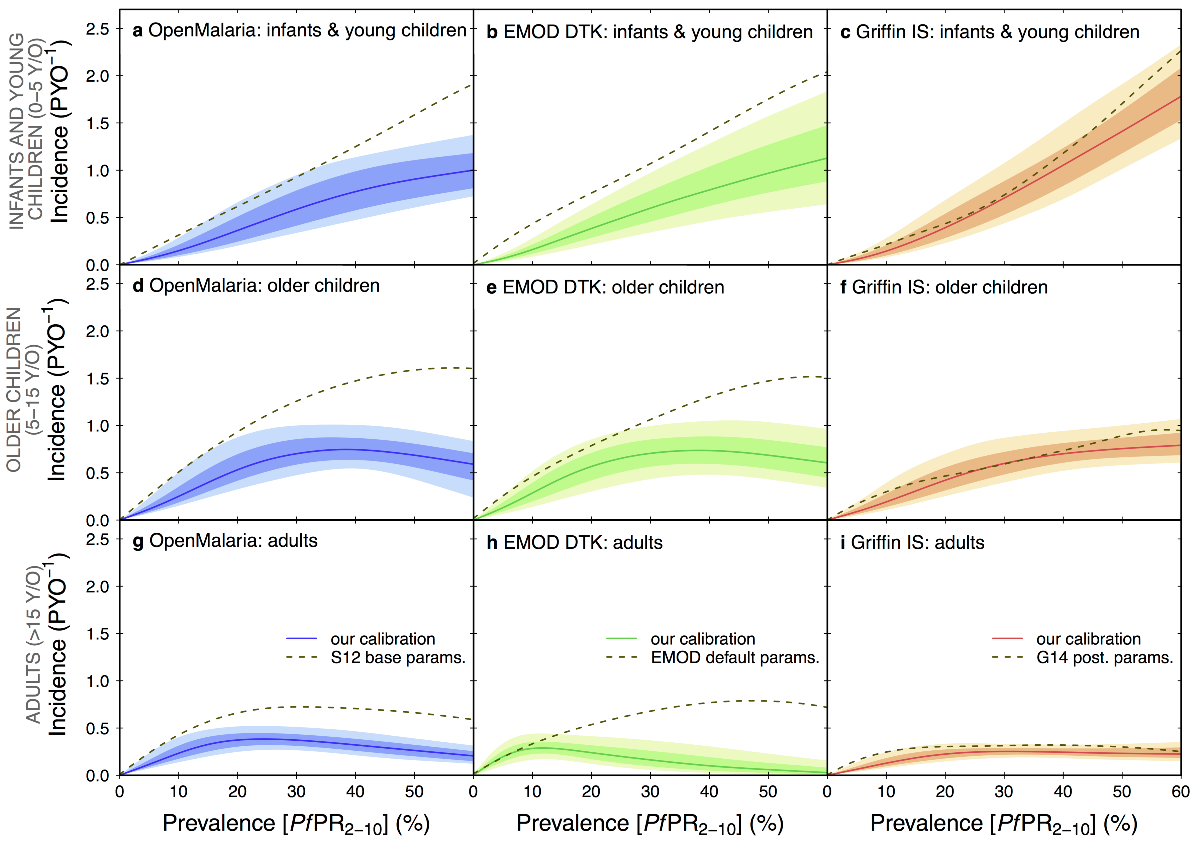


**Supplementary Figure 9: Comparison of our posterior predictions against previous calibrations of each the three "micro-simulation" codes comprising our ensemble for the *Plasmodium falciparum* prevalence–incidence relationship under conditions of low historical treatment and low transmission seasonality, stratified by age.** In each panel the coloured curve and shaded zones illustrate the (pointwise) median and surrounding 68% and 95% credible intervals for incidence detectable with daily ACD; while the broken black lines illustrate the equivalent outputs from our model emulator under fixed inputs corresponding to: **(a,d,g)** the "base model" parameterisation of OpenMalaria described by Smith *et al.*[2], **(b,e,h)** the default parameters for EMOD DTK, and **(c,f,i)** the posterior medians for steady-state fits of the Griffin et al. model[1].

## Supplementary Discussion

### Inspection of Age–Incidence Fits by Site

In Supplementary Figs. 3, 5, and 7, and Supplementary Figs. 4, 6, and 8, we present comparisons of our posterior fits to the age–incidence and age–prevalence observations, respectively, for each of the 24 studies (spilt into 32 site+year panels) in our benchmark dataset using our emulators for OpenMalaria, the EMOD DTK, and the Griffin IS. Consistent with the analysis of the Griffin *et al.*[1] model we find that although each code is able to broadly reproduce the age shift in burden as a function of transmission intensity, and hence underlying prevalence, there are numerous sites for which the data exhibit significant structural departures from the range of model-based age–incidence curves; hence, the need to allow for a large study-specific random effects term and extra-Poissonian variation in our likelihood function so as to avoid any single study having undue influence on the joint posterior.

### Comparison to Previous Work

Previous efforts to constrain each of the three transmission models in our ensemble have been markedly heterogeneous in terms of fitting methodology and datasets studied, and none has accounted for both seasonality as a random effect *and* the "observer effect" of study-based treatment. For reference it is therefore instructive to compare the *Pf*PR–incidence curves predicted under the optimum parameters of these previous fits against the results recovered here with our homogeneous calibration against a common benchmark dataset. In Supplementary Fig. 5 we illustrate the prevalence–incidence curves output by our emulator under a canonical parameterization of each micro-simulation model for the case of zero EIR seasonality and a low effective treatment rate of 35%: for OpenMalaria we use the "base model" fit reported by Smith *et al.*[2], for the Griffin IS we use the posterior medians reported by Griffin *et al.*[1], and for the EMOD DTK we use the default values suggested in the online user manual.

Given that the dataset previously fit by Griffin et al.[1] contains the majority of surveys included in the present study, it is no surprise that we recover a close agreement between our respective calibrations; although it is worth noting that the small differences seen here in our posterior predictives would likely propagate to differences of up to tens of millions of cases when employed for burden estimation at the continental level. Moreover, the close agreement of the adult *Pf*PR–incidence curves is as much due to the inflexibility of the Griffin model for this cohort as it is to the similarity of our fitted parameters. In contrast, our calibrations of the OpenMalaria and EMOD DTK codes have produced *Pf*PR–incidence curves markedly different to those predicted under their benchmark settings. For all age groups the OpenMalaria and EMOD DTK benchmark curves rise above ours at prevalences greater than 10% and exhibit a lower propensity towards declines at high prevalence.

## Supplementary References


1 Griffin, J. T., Ferguson, N. M. & Ghani, A. C. Estimates of the changing age-burden of Plasmodium falciparum malaria disease in sub-Saharan Africa. *Nature Comm.* **5**, 3136 (2014).

2 Smith, T. *et al.* Ensemble modeling of the likely public health impact of a pre-erythrocytic malaria vaccine. *PLoS Med.* **9**, e1001157 (2012).
